# Supplementary material for: New Intracellular Peptide Derived from Hemoglobin Alpha Chain Induces Glucose Uptake and Reduces Blood Glycemia
Source: Pharmaceutics. 2021 Dec 16;13(12):2175. doi: 10.3390/pharmaceutics13122175 (PMC8708875; doi:10.3390/pharmaceutics13122175)
Supplement: Supplementary file 1 [file pharmaceutics-13-02175-s001.zip › pharmaceutics-1491448-supplementary.pdf]

pharmaceutics

Supplementary Materials: New Intracellular Peptide Derived from Hemoglobin Alpha Chain Induces Glucose Uptake and Reduces Blood Glycemia

Renée N. O. Silva, Ricardo P. Llanos, Rosangela A. S. Eichler, Thiago B. Oliveira, Fábio C. Gozzo, William T. Festuccia and Emer S. Ferro

Table S1. Primers sequences.

Gene	Sequence	Amplicon (bp)	Accession number
Peroxisome Proliferator Activated Receptor Gamma (PPAR-γ)	Fwd: CATAAAGTCCTTCCCGCTGA Rev: GAAACTGGCACCCTT-GAAAA	102	NM_001127330.2
Peroxisome Proliferator Activated Receptor Alpha (PPAR-α)	Fwd: TGCAATTCGCTTTGGAA-GAA Rev: CTTGCCCAGAGATTT-GAGGT	118	NM_011144.6
Aldolase A, Fructose-bisphosphate (AldoA)	Fwd: GAGCTGTCTGACATCGCTCA Rev: TCTCGTGGAAGAGGATCACC	204	NM_001177307.1
Lipoprotein Lipase (LPL)	Fwd: GTCTGGCTGACACTG-GACAAA Rev: CCCAC-TTTCAAACACCCAAA	122	NM_008509.2
Phosphoglycerate Mutase 2 (Pgam2)	Fwd: TGGAATGAGGAGATCG-CACC Rev: ATTCCAGTGGG-CAGGTTCAG	143	NM_018870.3
Myosin, Light Chain 1 (MYL-1)	Fwd: GGAGGCATTTCTCCTGTTTG Rev: CCTGGTCCTTGTTGTTGGAG	133	NM_021285.3
Small Muscle Protein X-linked (SPMXc)	Fwd: CAGCCTCCCAGAAGGAAAG Rev: ACTGTTCACCTTTGGGGACA	113	NM_001252591.2
cAMP Responsive Element Binding Protein 1 (Creb1)	Fwd: GGTGCCAAGGATTGAA-GAAG Rev: GTACCCCATCCGTAC-CATTG	110	NM_133828.2
Cytochrome c Oxidase Subunit IV Isoform 1 (COX4i1)	Fwd: CGCTGAAGGAGAAGGA-GAAG Rev: GGATGGGGCCATACACATAG	77	NM_009941.3
Troponin I, skeletal, fast 2 (TNNI2)	Fwd: TGCAAACAACTG-CATGCGAA Rev: TTGAACTT-GCCCCTCAGGTC	131	NM_009405.3
Troponin I, skeletal, fast 3 (TNNT3)	Fwd: CCCCAGCCTTTCTCAGACTC	106	NM_001163664.1

	Rev: TTGGGCCTCCTCTTCCTCTT		
Insulin-like Growth Factor 1 (IGF1)	Fwd: TGTACTTCAGAA-GCGATGGGG<br>Rev: AGAGGTGTGAAGAC-GACATGA	112	NM_010512.5
Solute Carrier Family 2 - Slc2a4 (GLUT4)	Fwd: CCAACAGCTCTCAGGCATCA<br>Rev: CAGCTCCTATGGTGGCG-TAG	98	NM_001359114.1
Glyceraldehyde 3-phosphate de-hydrogenase (GAPDH)	Fwd: GTGCAG-TGCCAGCCTCGTCC<br>Rev: CAGGCGCCCAATAC-GGCCAA	75	BC_085275

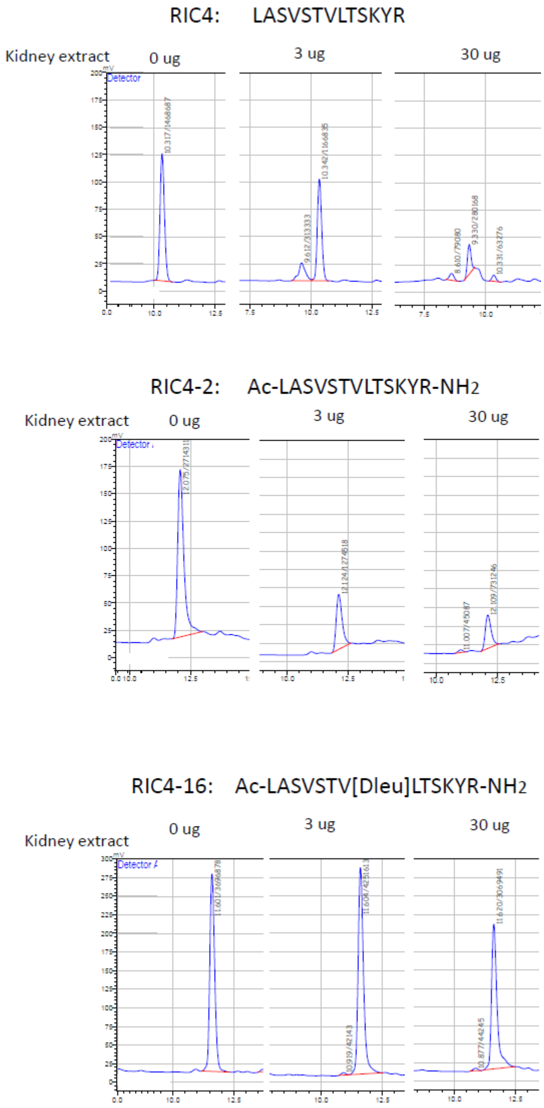

Figure S1. Typical experiments to evaluate the enzymatic stability of Ric4 and derivatives on kidney tissue extracts. In order to determine if the peptides investigated herein were substrates of tissue peptidases, 50 µM of each peptide was individually incubated for 20 min in the presence of increasing crude kidney tissue extracts (0 µg, 3 µg or 30 µg, as indicated), in a final volume of 250 µl of 0,025 M Tris-HCl, containing 0.125 M NaCl (TBS). Peptide hydrolyses were analyzed by reverse phase liquid chromatography (HPLC) using a C18 µBond-pak column (4.6 x 250 mm; Millipore Corp.) with a linear gradient of 5-65% acetonitrile in 0.1 % TFA for 20 min at a flow rate of 1 mL/min,

and absorbance monitored at a wavelength of 214 nm, as previously described [8,36,37]. Cleaved peptide bonds were identified by mass spectrometry sequencing after isolating the cleavage fragments manually after HPLC (data not shown). Note the greater stability of the Ric4-derivative Ac-LASVSTV[DLeu]TSKYR-NH2 (Ric4-16) compared to Ac-LASVSTVLTSKYR-NH2; Ric4-2 (Ric4-2) or LASVSTVLTSKYR (Ric4).
